# Marine Plankton-Derived Whitlockite Powder-Based 3D-Printed Porous Scaffold for Bone Tissue Engineering

**DOI:** 10.3390/ma15103413

**Published:** 2022-05-10

**Authors:** Ji-Won Baek, Ho Park, Ki-Su Kim, Sung-Kun Chun, Beom-Su Kim

**Affiliations:** 1 Department of R&BD, Cellco Inc., 208, Venture Startup Center, Jeonju University, 303, Cheonjam-ro, Wansan-gu, Jeonju-si 55069, Korea; bjw2151@cellco.co.kr (J.-W.B.); cellco8885@gmail.com (K.-S.K.); 2 Department of Clinical Laboratory Science, Wonkwang Health Science University, 514, Iksan-daero, Iksan-si 54538, Korea; bacoya@naver.com; 3 Department of Physiology, Jeonbuk National University Medical School, Jeonju-si 54907, Korea; sungkun.chun@jbnu.ac.kr; 4 Carbon Nano Convergence Tech Center, Jeonbuk National University, Jeonju-si 54896, Korea

**Keywords:** 3-dimensional printing, whitlockite, scaffold, bone, tissue engineering

## Abstract

Powder-based 3D printing is an excellent technique for the fabrication of complex structural shapes. The outstanding bone remodeling capacity of calcium phosphate bioceramics is a desirable characteristic for such fabrication. Whitlockite (WH) is a calcium phosphate-based ceramic that contains Mg ions and possesses good mechanical properties, rapid resorbability, and promotes osteogenesis. The aim of this study was to fabricate 3D-printed scaffolds using marine plankton-derived WH (MP-WH) powder. The surface morphology and composition of the fabricated scaffolds were characterized by scanning electron microscopy and X-ray diffraction. The biocompatibility and osteogenic effects were evaluated using human mesenchymal stem cells. We successfully obtained a 3D porous scaffold using MP-WH. The MP-WH 3D scaffold showed improved compressive strength compared to the tricalcium phosphate (TCP) 3D scaffold. The in vitro results showed that compared with TCP 3D scaffolds, MP-WH 3D scaffolds were biocompatible and enhanced cell proliferation and adhesion. In addition, alkaline phosphatase activity and real-time polymerase chain reaction assays demonstrated that osteoblast differentiation was improved on the MP-WH scaffold. These results suggest that marine plankton-derived WH is useful for fabricating 3D-printed scaffolds for bone tissue engineering applications.

## 1. Introduction

Critical-size bone defects can be caused by many conditions including trauma, tumors, and bone diseases. These critical-size bone defects cannot heal themselves and are considered to be of a size that requires secondary surgery using bone graft materials or scaffolds. Although the standard of the size in the range from 1–3 cm is controversial, Schemitsch et al. [1] explained that defects with a size of ≥2.5 cm have poor natural healing. Therefore, the application of artificial bone replacement has high clinical potential for bone defect filling [2] using scaffolds. In bone regeneration tissue engineering, three-dimensional (3D) scaffolds are required for tissue regeneration because they provide an environment for cell adhesion, proliferation, and differentiation [3,4]. Currently, the common method used to generate a 3D scaffold is the 3D printing (3DP) technique. This technique has been investigated as a useful tool to produce 3D scaffolds because it allows for control of the pore size, porosity, and outer shape of the scaffold [5]. In bone tissue engineering, 3DP-based ink-jet technology is considered one of the most future-oriented rapid prototyping (RP) systems. RP processes are well-established techniques used to manufacture prototypes based on computerized 3D data obtained using anatomical information derived from computed tomographic scan images of patients. This technique is a powder-based RP system based on the layer-by-layer principle of building 3D models [6]. During the printing process, the binder solution is jetted onto a pre-deposited thin powder. In the bone tissue engineering part of the RP system, several calcium phosphate materials are commonly used [7] for the preparation of scaffolds.

Among these materials, tricalcium phosphate (TCP, Ca_3_(PO_4_)_2_) bioceramic is frequently used as a scaffold material for bone due to its excellent bioactivity, osteoconductivity, compositional similarity to bone, and ability to directly bind to hard tissue [8,9]. In addition, TCP exhibits high biodegradability compared to hydroxyapatite (HAp), which is hardly degraded in the body, and its higher solubility is advantageous in many applications [10]. However, the poor osteoinductive properties of TCP are still limiting factors for clinical application [11].

In addition, when designing a scaffold for bone tissue engineering, the compressive strength of the scaffold is considered an influencing parameter for load bearing defects. Although the compressive strength of calcium phosphate-based ceramic 3D scaffolds can be increased by the sintering process [12], this single process remains insufficient to improve the compressive strength of scaffolds.

Whitlockite (WH) is a calcium phosphate-based ceramic containing magnesium ions, the second most abundant mineral in the human bone. It accounts for about 25–35 wt% of the minerals in the human bone, and its solubility in the body is higher than that of HAp. In particular, WH has higher mechanical compressive strength than that of HAp and TCP, and the continuous release of magnesium and phosphate ions causes high expression of osteogenic genes and promotes bone growth, making it an excellent bone regeneration material [13,14,15,16,17].

In this study, we first prepared marine plankton-derived WH (MP-WH) powder and then fabricated a highly porous 3D scaffold through 3DP and sintering. The surface morphology and chemical properties of the fabricated 3D scaffolds were characterized by scanning electron microscopy (SEM) and X-ray diffraction (XRD) analysis. The improved mechanical strength due to Mg contained in MP-WH was evaluated by comparing the compressive strength with the TCP scaffold. The biological behavior of the 3D scaffold was then investigated in vitro for bone tissue engineering.

## 2. Materials and Methods

### 2.1. Powder Preparation and 3D Printing

**Figure 1:**
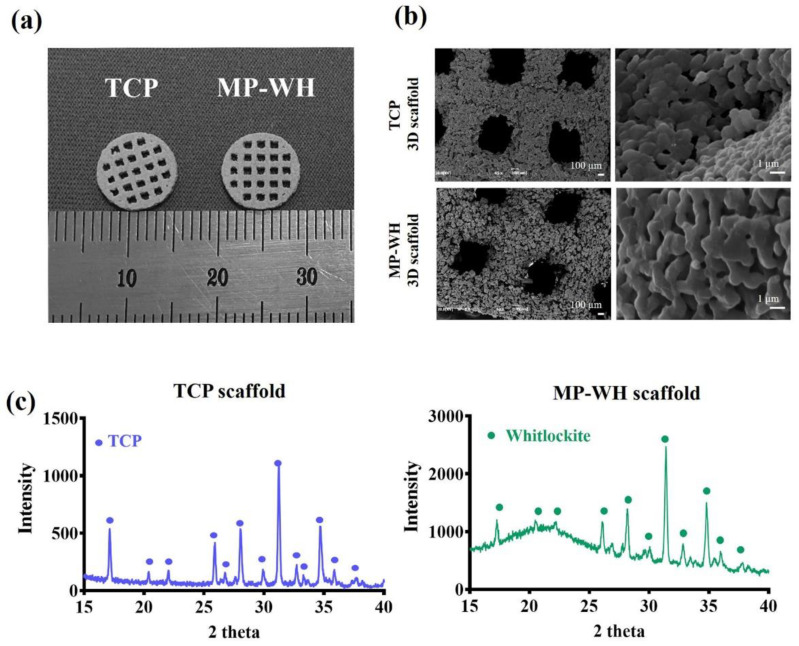
Fabricated open porous 3D scaffold using the 3D printing machine (**a**) and scanning electron microscope image of the 3D scaffold (**b**). X-ray diffraction analysis (**c**) of the fabricated definitive 3D scaffold. TCP, tricalcium phosphate; MP-WH, marine plankton-derived whitlockite.

Marine plankton-derived whitlockite (MP-WH) powder was synthesized using the exoskeleton of foraminifer via a hydrothermal reaction method. Briefly, raw foraminifer exoskeletons were washed and dried at 60 °C. The foraminifer’s exoskeletons were converted into WH using a hydrothermal reaction based on a previously established method [18]. After synthesizing MP-WH, the powder was ball-milled and sieved to <20 μm. Tricalcium phosphate (TCP) powder (Sigma Aldrich, St. Louis, MO, USA) with an average particle size of 10–20 μm was used. To fabricate the 3D scaffold, a porous square-shaped structure (10 × 10 × 10 mm) was designed using a 3D modeling program (SolidWorks, SolidWorks Corp., Concord, CA, USA). The pore channel structure had a square cross-section of 0.5 × 0.5 mm^2^ and the channel was 100% interconnected. The 3D scaffold was printed using a 3DP equipment system (Z350, Z-Corporation, Burlington, NJ, USA) (Figure 1a) under a 100 μm layer thickness condition. For the 3D printing layer, water-based phosphoric acid (10% *v*/*v*) was used as the binder solution. After building, all specimens were left in the machine for 12 h and then air blown to remove any unbound powder. The fabricated 3D scaffolds were heated at 300 °C for 30 min to remove the binding solution. Finally, the scaffolds were sintered at 900 °C for 72 h and 3D scaffolds were obtained.

### 2.2. Micro-Structural Observation and X-ray Diffraction Analysis

To evaluate the microstructure and surface morphology of the 3D scaffold, the samples were sputter coated with gold for 3 min under vacuum and observed using a scanning electron microscope (SEM, EM-30; COXEM, Daejeon, Korea). To confirm the composition of the 3D scaffold, X-ray diffraction (XRD) analysis was performed with CuKα radiation at a scanning rate of 0.02°/min (2θ) and a 30 mÅ range of 15–40°.

### 2.3. Determination of Heavy Metal Content and Trace Elements

The concentrations of heavy metals and trace elements were evaluated using inductively coupled plasma–optical emission spectrometry (ICP-OES). Briefly, MP-WH powder was dissolved in a mixed solution of nitric acid and hydrochloric acid. The sample solution was then sprayed through argon plasma for measurement of individual elements.

### 2.4. Compressive Strength and Porosity Analysis

The compressive strength was evaluated using an Instron test machine (Model 4505; High Wycombe, UK) by applying a load via a 1 N load cell at a crosshead speed of 0.5 mm/min. Scaffold porosity was evaluated using a mercury intrusion porosimeter (AutoPore IV9500; OakRidge, TN, USA). Briefly, the scaffolds were sealed in a penetrometer, weighed, and subjected to analysis [19].

### 2.5. Cell Culture

Human mesenchymal stem cells (hMSCs) (ATCC, Manassas, VA, USA) were used to evaluate the biocompatibility of the 3D scaffolds. Cells were maintained at 37 °C in a 5% CO_2_ incubator using 10% fetal bovine serum supplemented with Dulbecco’s modified Eagle medium (Gibco-BRL, Gaithersburg, MD, USA) media.

For 3D culture on scaffolds, the scaffold was sterilized with 70% alcohol and rinsed three times with 1× phosphate-buffered saline (PBS). Cells (5 × 104 cells/well) were seeded on each scaffold. After seeding, the cell constructs were incubated for 1 h to allow for initial cell adhesion to the surface and were transferred to a new culture dish. At various time intervals, the constructs were used for cell proliferation, Live/Dead^®^ assay, cell adhesion, and osteoblast differentiation analyses.

### 2.6. Cell Proliferation Assay

hMSCs (1 × 10^5^ cells) were seeded and cultured on TCP and MP-WH 3D scaffolds. The colorimetric 3-(4,5-dimethylthiazol-2-yl)-5-(3-carboxymethoxyphenyl)-2-(4-sulfophenyl)-2H-tetrazolium) (MTS) assay was performed at predetermined times (1, 5, and 10 days). In this study, the CellTiter96^®^ kit (Invitrogen, Carlsbad, CA, USA) was used for the MTS assay. Briefly, 200 µL of MTS reagent was mixed with 1 mL of medium and added to each well. After 2 h of incubation, the supernatant was collected and absorbance was measured at 490 nm using an ELISA reader (SpectraMAX M3; Molecular Devices, Sunnyvale, CA, USA). A DNA content assay was performed to confirm cell density.

### 2.7. Viability/Cytotoxicity Assay

A fluorescence-based Live/Dead^®^ kit (Molecular Probes, Eugene, OR, USA) was used to assess cell viability and cytotoxicity. Briefly, hMSCs were seeded and cultured for 2 days. The cell constructs were then rinsed with PBS. The prepared working solutions of calcein acetoxymethyl ester and ethidium homodimer-1 were added to each well and incubated for 30 min in a CO_2_ incubator. Fluorescence images were obtained using a fluorescence microscope (DM IL LED Fluo; Leica Microsystems, Wetzlar, Germany).

### 2.8. Observation of Cell Adherence

To evaluate cell attachment and growth on the scaffolds, hMSCs were seeded onto each scaffold, cultured for 5 days, and observed by SEM. After incubation, the cell-constructed scaffolds were washed with PBS, fixed in 2.5% glutaraldehyde solution at 4 °C for 1 h, and post-fixed with 0.1% osmium tetroxide solution for 30 min at 25 °C. Dehydration processing was performed using a graded ethanol series (25%, 50%, 75%, 95%, 100%, and 100%). The dehydrated samples were sputter coated with gold and observed using SEM (EM-30).

### 2.9. Alkaline Phosphatase Activity Assay

To determine osteoblast differentiation, the alkaline phosphatase (ALP) activity assay was performed as described previously [20]. Briefly, cells were seeded and cultured in media containing an osteoblast differentiation reagent for 7 days. The cultured scaffolds were rinsed with PBS and adherent cells were dissolved from the scaffold using 1% Triton X-100/PBS solution. The samples were chopped into small pieces and centrifuged at 14,000× *g*. After centrifugation, the supernatant was used for ALP activity assay. Total protein content was determined using a BCA protein assay kit (Pierce, Rockford, IL, USA) and used for normalization of ALP activity.

### 2.10. Real-Time Polymerase Chain Reaction

The mRNA expression of several osteoblast marker genes was assessed to determine osteoblast differentiation using quantitative real time polymerase chain reaction (qRT-PCR). Briefly, hMSCs were seeded and cultured. After 7 days of cultivation, total mRNA was extracted using an RNA isolation kit (Ribospin, GeneAll, Seoul, Korea). PCR was performed using the TaqMan Universal PCR Master Mix, TaqMan primers, and probe sets specifically targeting ALP (Hs01029144_m1), collagen type 1 α I (Col1αI) (Hs00164004_m1), Runx2 (Hs00231692_m1), osteocalcin (OC) (Hs01587814_g1), and 18S rRNA (Hs99999901_s1) (Applied Biosystems, Carlsbad, CA, USA). The 18S rRNA gene was used as the internal standard. The relative expression was normalized to that of the TCP scaffold.

### 2.11. Statistical Analysis

All experiments were performed in triplicate. Values are expressed as the mean ± standard deviation (SD). Statistical analysis was performed using the Student’s *t* test and one-way analysis of variance; *p* < 0.05 was considered statistically significant.

## 3. Results

### 3.1. Fabrication of 3D Printing Model

First, a porous 3D scaffold was fabricated using TCP powder and synthesized MP-WH powder using a Z350 3D printing machine (Z350; Z Corporation, MA, USA). The printed scaffold was successfully fabricated with open channels based on 3D computer modeling (Figure 1a), and SEM images of the surface of the 3D scaffold showed pores of the TCP and synthesized MP-WH powders approximately 1 mm in size in accordance with the designed macropore structure. The high-magnification SEM image showed similar particle morphologies after sintering. TCP and MP-WH scaffolds presented structures with well-sintered agglomerated spherical or quasi-spherical particles with homogeneous microporosity (Figure 1b). XRD was used to characterize the crystal structures of the TCP and MP-WH scaffolds. The XRD results showed peak intensities of TCP scaffolds according to the powder diffraction pattern of the *β*-TCP ICSD card. Similarly, the MP-WH scaffolds showed whitlockite peaks in the powder diffraction pattern (Figure 1c). These results suggest that the fabrication process did not alter the crystal structure of the powder.

### 3.2. Compressive Strength and Porosity

**Figure 2:**
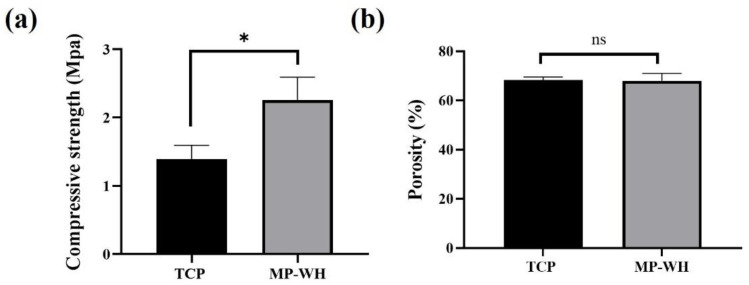
Compressive strength (**a**) and porosity (**b**) of tricalcium phosphate (TCP) and marine plankton-derived whitlockite (MP-WH) 3D scaffolds. Data are represented as the mean ± standard deviation (SD) for three samples. * indicates significant differences when compared with the TCP 3D scaffold (*p* < 0.05). ‘ns’ indicates not significant.

The compressive mechanical testing results demonstrated the mechanical strength of the 3D scaffolds. The compressive strengths of the TCP scaffold (1.39 ± 0.2 MPa) and MP-WH scaffold (2.26 ± 0.33 MPa) were statistically different. (Figure 2a). The total porosity results showed that the total porosities of the TCP (68.2 ± 0.7%) and MP-WH (68.0 ± 1.7%) scaffolds were not significantly different (Figure 2b).

### 3.3. ICP-OES Analysis

The ICP-OES analysis showed that heavy metals such as As, Pb, Cd, and Hg were not detected in the MP-WH powder. In contrast, MP-WH contained several trace elements, such as Mg, K, Si, and Sr (Table 1).

### 3.4. Cell Proliferation Cultured on the 3D Scaffold

**Figure 3:**
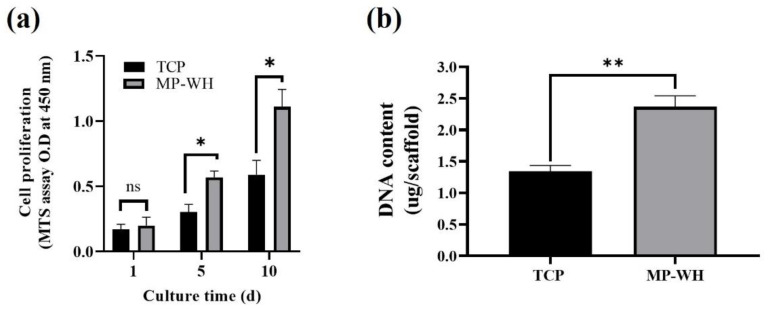
Cell proliferation measurement using the MTS (**a**) and total DNA content (**b**) assays. Human mesenchymal stem cells (hMSCs) were cultured on 3D and infiltrated scaffolds, and the MTS assay was performed. DNA content was measured after 5 days of cultivation. Data are represented as the mean ± standard deviation (SD) for three samples. * *p* < 0.05 and ** *p* < 0.01 indicate significant differences when compared with the cells cultured on the tricalcium phosphate (TCP) 3D scaffold. MP-WH, marine plankton-derived whitlockite. ‘ns’ indicates not significant.

To determine the proliferation of cells grown on each 3D scaffold, the mitochondrial activity-based MTS assay was used. The results of the MTS assay showed that hMSCs grew well over time, and the optical density values of the MP-WH 3D scaffold were similar to those of the TCP 3D scaffold after 1 day of cultivation. However, after 5 and 10 days of cultivation, the cell growth rate rapidly increased on the MP-WH 3D scaffold compared with that on the TCP scaffold (Figure 3a). To confirm cell proliferation, the total DNA content was evaluated after 5 days. Consistent with the MTS assay results, the analysis results of total DNA content (Figure 3b) showed that the density of cultured cells on the MP-WH scaffold (2.36 ± 0.17 μg) was higher than on the TCP scaffold (1.34 ± 0.09 μg).

### 3.5. Cell Viability and Cytotoxicity Assay Cultured on the 3D Scaffold

**Figure 4:**
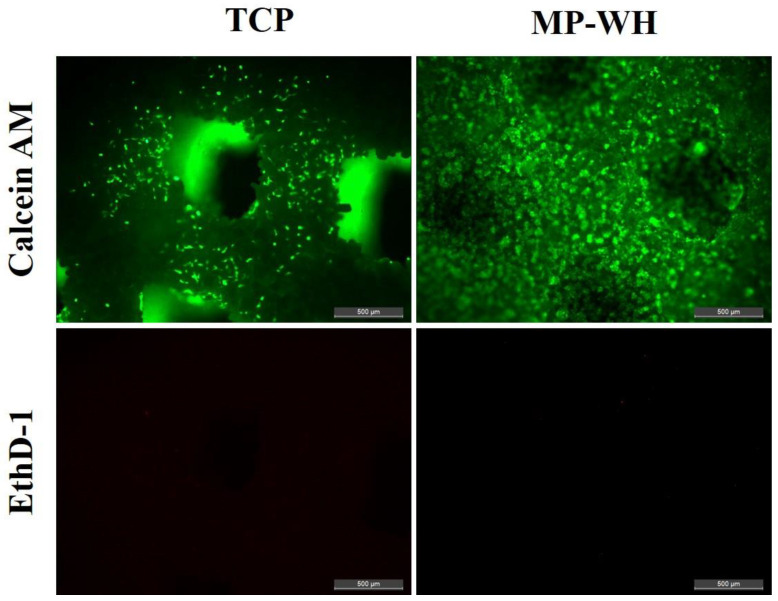
Cytotoxicity evaluation of the fabricated 3D scaffold. Viability/cytotoxicity fluorescence imaging was performed 2 days after seeding on tricalcium phosphate (TCP) and marine plankton-derived whitlockite (MP-WH) 3D scaffolds. Calcein acetoxymethyl (Calcein AM)-stained healthy cells appear as green, and ethidium homodimer-1 (EthD-1)-stained nuclei of dead cells appear as red. Scale bar: 500 μm.

To evaluate the cytotoxicity of the fabricated TCP and MP-WH 3D scaffolds, fluorescence live/dead staining assays were performed (Figure 4). The fluorescence image results showed that most of the hMSCs were viable (stained green), and no dead cells (red dots) were observed. These results indicate that the fabricated 3D scaffolds were not associated with cytotoxicity. In addition, the number of adherent live cells (density of green dots) was higher on the MP-WH scaffold than on the TCP scaffold.

### 3.6. Cell Adhesion Observation by SEM

**Figure 5:**
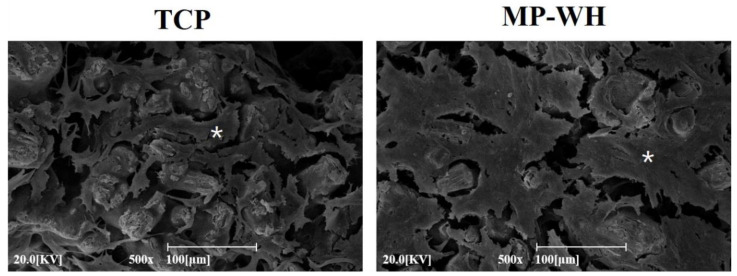
Scanning electron microscopy images of human mesenchymal stem cells (hMSCs) cultured on tricalcium phosphate (TCP) and marine plankton-derived whitlockite (MP-WH) 3D scaffold. Cells were seeded on TCP and MP-WH scaffolds and cell adhesion and growth were observed after 5 days of cultivation. * indicates cell growth on the scaffold.

To evaluate cell attachment, SEM analysis was performed after 5 days of cultivation. The SEM micrographs (Figure 5) show that hMSCs adhered well and grew on the surfaces of the TCP and MP-WH scaffolds. The cells grew on the scaffold surface, formed cell layers, and spread on the surface of the scaffold. In particular, the adherent cell density was higher on the MP-WH 3D scaffold than on the TCP scaffold (Figure 5).

### 3.7. Measurement of Osteoblast Differentiation

**Figure 6:**
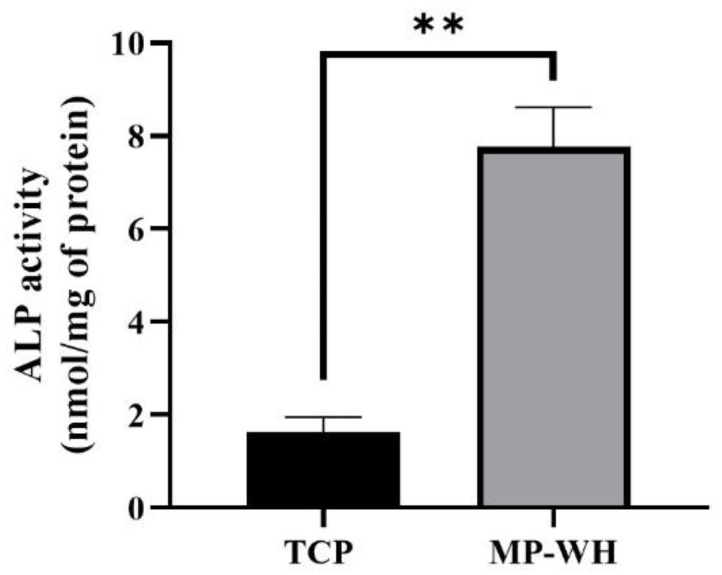
Alkaline phosphatase (ALP) activity of human mesenchymal stem cells (hMSCs) cultured on tricalcium phosphate (TCP) and marine plankton-derived whitlockite (MP-WH) 3D scaffold. Data represent the mean ± standard deviation (SD) for three samples. ** *p* < 0.01 indicates significant differences from that of the TCP 3D scaffold.

**Figure 7:**
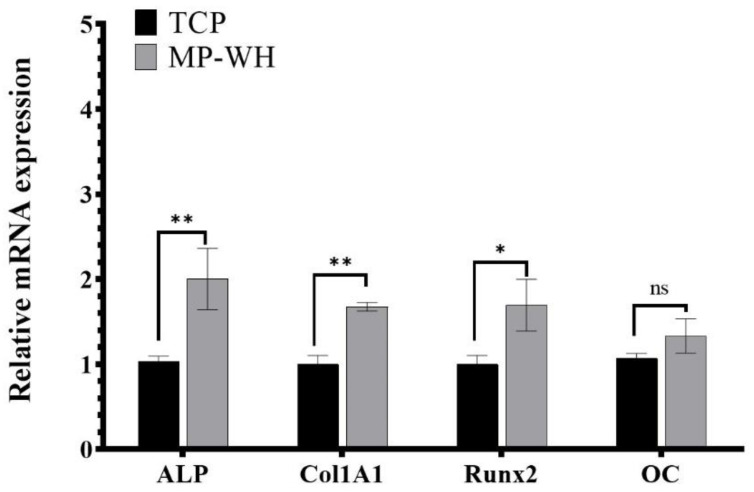
Relative mRNA expression of osteoblast marker genes by quantitative real-time polymerase chain reaction (qRT-PCR). Expression of each osteoblast marker gene is normalized to that of 18S rRNA, and the relative expression level was normalized to that of cells cultured on the tricalcium phosphate (TCP) 3D scaffold. Data represent the mean ± standard deviation (SD) for three samples. * *p* < 0.05 and ** *p* < 0.01 indicate significant differences from that of the TCP scaffold. ALP, alkaline phosphatase; MP-WH, marine plankton-derived whitlockite. ‘ns’ indicates not significant.

To determine whether the MP-WH 3D scaffold supported differentiation into osteoblasts, hMSCs were cultured on the TCP and MP-WH scaffolds. ALP activity and qRT-PCR analyses were performed to detect the expression of osteoblast markers. The ALP activity significantly increased in cells cultured on the MP-WH 3D scaffold (2.8 ± 0.3 nmol/mg of protein) than on the TCP scaffold (1.4 ± 0.1 nmol/mg of protein) (Figure 6). Furthermore, to confirm the osteogenic activity of the scaffold, several osteoblast differentiation marker genes were evaluated after 7 days of cultivation. The qRT-PCR results for ALP were consistent with the ALP activity results. Furthermore, the mRNA expression levels of Col1A1 and Runx2 were significantly higher in cells cultured on the MP-WH scaffold than those cultured on the TCP scaffold. However, although the mRNA expression of OC was slightly increased in cells cultured on the MP-WH scaffold, it was not significantly different between cells on the MP-WH TCP and TCP scaffolds (Figure 7).

## 4. Discussion

Ceramic-based 3D-printed scaffolds for bone tissue engineering allows for the fabrication of scaffolds that mimic the porous architecture and material composition of the trabecular bone. Bioceramics such as HAP have been used to fabricate scaffolds using the 3DP technique. Calcium phosphate-based ceramic powders, such as HAp and *β*-TCP, are beneficial for bone tissue regeneration owing to their exceptional biocompatibility, bioactivity, and osteoconductivity. HAp and β-TCP can be synthesized through chemical methods using various precursors of calcium and phosphate ions. However, chemically prepared stoichiometric HAp and β-TCP do not contain trace elements (Mg, Sr, etc.) that promote osteoblast adhesion and bone formation, unlike pure HAp and β-TCP [21,22]. To overcome this, many studies have been conducted on bioceramic materials obtained from natural sources similar in chemical composition to that of the bone tissue for excellent bone regeneration. Since HAp or β-TCP extracted from natural sources such as egg shells [23], snail shells [24], fish bones [25], and corals [26] contain trace elements, they exhibit superior cell proliferation and promote differentiation than the stoichiometric HAp or β-TCP do.

In this study, we synthesized Mg-containing β-TCP, Whitlockite (WH), using marine plankton as a natural source. Using marine plankton from the ocean, a rich source of materials with biological potential, could be a significant advantage in the field of biomaterial development. We focused on the fabrication of a 3D porous scaffold using synthesized WH powder and its bioactivity with a TCP scaffold in vitro.

In the present study, a 3D porous scaffold was fabricated using marine plankton-derived WH powder using an RP machine. The pore size considered when designing the 3D model was affected by the scaffold properties related to cell adhesion, migration, and formation of new blood vessel. Previous reports suggested that a pore size of 0.5 mm or 1–2 mm of the scaffold for bone regeneration is effective for new bone formation [27,28]. Therefore, in this study, based on the results of previous studies, a scaffold with a 1 mm pore size was designed considering the size, adhesion, growth of osteoblasts, and resolution of the 3DP equipment system. According to the SEM images, compared with the designed 3D model, the printed scaffolds showed high printing fidelity, including that of the overall shape and pore size. The XRD results also showed that the 3D scaffold manufactured using TCP powder comprised TCP peaks, and the scaffold manufactured using whitlockite powder was also consistent with the whitlockite peak pattern. These results indicate that all the components used as binder solution compositions were completely removed through sintering at 900 °C. Furthermore, these results showed that the fabrication process did not alter the crystal structure of the raw powder materials.

In bone tissue engineering, scaffolds occasionally require sufficient mechanical strength for load bearing. Therefore, compressive strength is considered to be an important factor. To fabricate the 3D scaffold, a binding solution was used to bond the powder to the lamination of the layer, and the binder solution was removed through the sintering process. In addition, according to the high-magnification SEM image, the surface of the particles exhibited a spherical shape aggregated with each other according to sintering at 900 °C. The compressive strength of the TCP scaffold was approximately 1.6 MPa, which corresponds to the minimal strength of the human cancellous bone. However, when MP-WH was used under the same manufacturing conditions, the compressive strength slightly improved compared to that of the TCP scaffold. The raw material of marine plankton contains Mg and other trace elements. Therefore, MP-WH also contains trace elements including Mg [29]. Khorashadizade et al. [30] reported that HAp ceramics containing Mg had a higher mechanical strength than that of HAP ceramics that did not contain Mg. This is owing to the crystal structure according to the atomic arrangement of the ceramic powder, which differs based on the presence or absence of Mg [30]. Therefore, the increased compressive strength of the MP-WH scaffold may be attributed to the crystal structure of the WH material.

Porosity is also an important factor in biocompatibility because it can affect cell ingrowth [31]. In particular, in the regeneration of bone tissue, the porous structure is involved in the angiogenesis of blood vessels and the attachment of bone cells. In this study, the pore size was modeled as 1 mm, and a scaffold with an interconnected porous structure was prepared accordingly. The fabricated scaffold showed a porosity that was slightly less than approximately 70% in TCP and MP-WH and was not significantly different between the two. Therefore, the pore size and porosity are considered suitable for use in scaffolds for bone regeneration.

Biocompatibility testing is a starting point for the development of biomaterial scaffolds. In this study, we examined the proliferation, attachment, and differentiation of hMSCs because MSCs are multipotent cells that can differentiate into many types of cells, including osteoblasts [32]. No significant cytotoxicity was observed in any scaffold. In addition, cell proliferation was significantly increased on the MP-WH scaffold compared to the TCP scaffold. Furthermore, the cell density was continuously higher until 10 days on the MP-WH scaffold than on the TCP scaffold. The SEM images of cell attachment showed that a larger number of cells were attached and grown on the MP-WH scaffold than on the TCP scaffold.

WH is a calcium phosphate ceramic that contains Mg ions. Mg ions have been shown to promote cell proliferation and adhesion [17]. Under physiological conditions, Mg ions are released from WH and these ions then stimulate cell proliferation [33]. Mg ions released from WH significantly stimulated osteoblast cell proliferation [34] and bone formation in an in vivo animal model [35]. Therefore, these cell proliferation and growth results may be because of the Mg ions, which are present in MP-WH.

ALP activity is one of the most commonly used markers of osteoblastic differentiation. Therefore, ALP activity was analyzed to determine osteogenic activity. Col1αI also plays an important role [36,37] and Runx2 is an important transcription factor [38] during osteoblast differentiation. OC is a well-known osteoblast marker gene. Therefore, in this study, the mRNA expression levels of ALP, Col1αI, Runx2, and OC were evaluated using qRT-PCR. When hMSCs were cultured on each 3D scaffold, the ALP activity was significantly higher on the MP-WH scaffold than on the TCP scaffolds. Similar to the results obtained for ALP activity, the expression levels of Col1αI, Runx2 and OC were also significantly increased in cells cultured on coated MP-WH scaffolds.

Osteoblast differentiation is affected by intracellular communication and cell confluency [39]. Cell proliferation, DNA content, and cell attachment results showed that a higher cell confluence was easily reached the MP-WH scaffold culture owing to the possibility of the biological proliferation effect of the Mg ion [40]. This may also be because of the higher cell density of the scaffold and an increase in the amount of differentiation. The ICP-OES results showed that MP-WH contained abundant amounts of several types of trace elements such as Mg, Si, and Sr. Trace elements, such as Si and Sr ions, can also affect osteoblast differentiation [41]. Mg ions play an essential role in cell adhesion via the binding interaction of the integrin family of cell surface receptors and ligand protein [40]. Mg and Sr ions directly promote osteoblast differentiation [42,43] and bone formation [44,45]. Although we did not evaluate the signaling pathways of Mg and Sr ions on the cellular response, these previous findings indicate their role in the higher cell proliferation, adhesion, and osteoblast differentiation results with the MP-WH scaffold than with the pure TCP scaffold. These osteogenic activity results suggest that the 3D scaffold using marine plankton-derived WH ceramic supports the environment for osteoblast differentiation more than the pure TCP ceramic powder does.

## 5. Conclusions

In summary, this study demonstrated the possibility of using a 3D porous scaffold with MP-WH powder. MP-WH powder was obtained by hydrothermal reaction of raw foraminiferal exoskeletons. MP-WH has increased the clinical applicability of 3D porous scaffolds by taking advantage of the abundant marine resources and chemical composition similar to bone tissues. This MP-WH powder was successfully fabricated as a 3D scaffold using the RP printing method, and it was confirmed that the compressive strength was improved by MP-WH. In addition, the results of ICP-OES analysis confirmed the presence of trace elements, such as Mg and Sr ions, and the absence of heavy metals, confirming the safety of MP-WH scaffold as a biomaterial. The bioactivity of the trace elements contained in the MP-WH scaffold was revealed through the evaluation of cell proliferation, cytotoxicity, cell adhesion, and osteoblast differentiation. Furthermore, the results of in vitro tests using MP-WH scaffolds showed superior biocompatibility and osteogenic differentiation capacity versus pure TCP scaffolds did. Therefore, these results suggest that MP-WH has potential as a useful ceramic powder for 3D-printed scaffolds to enhance bone regeneration.

## Figures and Tables

**Table 1:** Heavy metal and trace element amounts in marine plankton-derived whitlockite determined by inductively coupled plasma–optical emission spectrometry (ICP-OES) analysis (unit: ppm).

Element	As	Cd	Hg	Pb	Ca	K	Mg	P	Si	Sr
	ND	ND	ND	ND	346,476	3795	32,854	198,798	99	1869

ND, not detected.

## Data Availability

Not applicable.
